# The Imipridone ONC206 Inhibits Tumor Growth and Improves Survival in Patient-Derived Xenograft Models of Uveal Melanoma

**DOI:** 10.3390/cancers18121895

**Published:** 2026-06-10

**Authors:** Mir Mustafa Ali, Md Alauddin, Iqbal Mahmud, Aron Joon, Aalim B. Momin, Jacob R. Cortez, Huiqin Chen, Lin Tan, Waikin Chan, Rachel William Anantha, Danielle L. Stolley, Diana Shamsutdinova, Kurt Evans, Funda Merric-Bernstam, Meenhard Herlyn, Monzy Thomas, Yeqing Chen, Michael A. Davies, Chandrani Chattopadhyay

**Affiliations:** 1 Department of Melanoma Medical Oncology, The University of Texas MD Anderson Cancer Center, 1515 Holcombe Blvd., Unit 362, Houston, TX 77030, USA; 2 Department of Bioinformatics & Comp Biology, The University of Texas MD Anderson Cancer Center, Houston, TX 77030, USA; 3 Department of Biostatistics, The University of Texas MD Anderson Cancer Center, Houston, TX 77030, USA; 4 Department of Neurosurgery, The University of Texas MD Anderson Cancer Center, Houston, TX 77030, USA; 5 Department of Metabolomics Core Facility, The University of Texas MD Anderson Cancer Center, Houston, TX 77030, USA; 6 Department of Hematopoietic Biology & Malignancies, The University of Texas MD Anderson Cancer Center, Houston, TX 77030, USA; dstolley@mdanderson.org; 7 Department of Translational Molecular Pathology, The University of Texas MD Anderson Cancer Center, Houston, TX 77030, USA; ddshamsutdinova@mdanderson.org; 8 Department of Investigational Cancer Therapeutics, The University of Texas MD Anderson Cancer Center, Houston, TX 77030, USA; 9 Melanoma Research Center, Molecular and Cellular Oncogenesis Program, The Wistar Institute, Philadelphia, PA 19104, USA

**Keywords:** uveal melanoma (UM), liver metastasis, oxidative phosphorylation, imipridones, PDX, autophagy, lipidomics

## Abstract

**Background/Objectives:** Uveal melanoma is the most common primary ocular cancer in adults. Patients with metastatic uveal melanoma (mUM) have limited treatment options and poor prognosis. mUM is characterized by high oxidative phosphorylation (OXPHOS), which may be a therapeutic vulnerability for this disease. ONC206 is an imipridone compound that can inhibit OXPHOS indirectly and is currently being evaluated in clinical trials. Thus, we tested the effects of ONC206 on human uveal melanoma cell lines and patient-derived xenografts (PDXs) in vitro and in vivo. **Methods:** The effects of ONC206 on cell survival, apoptosis, autophagy, oncogenic signaling pathways, and metabolic networks were assessed in vitro using human melanoma cell lines. ONC206 was then tested for safety and anti-tumor activity in vivo using two mUM PDX models. **Results:** ONC206 treatment produced dose-dependent inhibition of mUM cell growth in vitro, with induction of varying levels of apoptosis and autophagy. ONC206 treatment also downregulated OXPHOS effector proteins and metabolites, thereby impairing mitochondrial OXPHOS. Treatment with ONC206 significantly reduced tumor burden and improved survival in two UM PDX mouse models in vivo. **Conclusions:** Our findings position ONC206 as a mechanistically distinct agent to target mitochondrial metabolism and to inhibit mUM. As ONC206 is currently being evaluated in multiple clinical studies, our data support further evaluation as a potential new therapeutic strategy for mUM.

## Simple Summary

Patients with metastatic uveal melanoma (UM), the most common eye cancer in adults, have poor outcomes and few treatment options. Our previous studies demonstrated that high oxidative phosphorylation (OXPHOS) activity is associated with poor prognosis in this disease. We also showed that preclinical small molecules from the imipridone family can indirectly inhibit OXPHOS and reduce UM growth in vitro and in vivo. In this study, we tested the molecular and anti-tumor effects of ONC206, an imipridone that is currently being evaluated in clinical trials, in UM cell lines and patient-derived xenograft (PDX) models. Our studies show that ONC206 inhibits tumor growth and prolongs survival in mice with UM PDX. These results suggest that ONC206 could be a promising new therapy for patients with advanced UM.

## 1. Introduction

UM is the most common primary eye malignancy in adults. UM is associated with poor prognosis, as approximately half of the patients develop distant metastasis (mUM), predominantly in the liver. Currently, there are only two approved therapies for mUM: Tebentafusp and intra-arterial hepatic infusion of Melphalan. The use of Tebentafusp is limited to HLA-A*2:01-positive patients and achieves clinical responses in <10% of patients [1]. Intra-arterial hepatic perfusion of melphalan achieves higher response rates (~50%), but its activity is limited to liver lesions and the treatment has a high risk of procedure-related complications (20–40%) and morbidity (30-day mortality rates of 1–5%) [2,3].

Focal radiotherapy remains the standard of care, although recurrence is nearly universal. Surgical resection is rarely feasible, and systemic chemotherapy has not demonstrated meaningful survival benefit [4,5,6]. Though PD-1 inhibitors have demonstrated promising results in cutaneous melanoma, responses in metastatic UM are limited [7,8]. Recent studies have refined the molecular understanding of UM by linking genetic variation, mutation-associated expression profiles, inherited risk, chromosomal and epigenetic alterations, and tumor–immune interactions to disease susceptibility and progression [9,10,11,12]. Therefore, the development of more effective treatments remains a critical need for this disease.

Our previous studies demonstrated that UM is characterized by high OXPHOS (compared to 33 other cancers), implicating this metabolic pathway as a potential therapeutic target in this disease [13]. Since a direct inhibitor of OXPHOS caused intolerable toxicities in clinical studies [14], we have tested imipridones (ONC201, 206 and 212), a group of small molecules that activate mitochondrial protease CLPP [15], to degrade OXPHOS effectors and indirectly inhibit OXPHOS. Supporting the feasibility of this approach, ONC201, the first imipridone to be evaluated in patients, was recently approved by the US Food and Drug Administration (FDA) for the treatment of recurrent H3K27M-mutant diffuse midline glioma based on its activity and safety in patients [16]. We previously showed that ONC212, a more potent imipridone, reduced tumor growth and improved survival in orthotopic UM liver metastasis mouse models [17]. However, as ONC212 is currently in pre-clinical development and has not been evaluated in patients, here we evaluated ONC206, an imipridone with greater potency than ONC201 that is currently under evaluation in multiple clinical trials (NCT04541082, NCT04732065), in UM cell lines in vitro and PDX models in vivo.

Our studies here demonstrate that ONC206 treatment markedly suppresses OXPHOS activity, downregulates key OXPHOS effector proteins and metabolites, and induces apoptosis and/or autophagy in multiple human UM cell lines. Metabolomic profiling indicates that ONC206 induces systemic alterations in cellular metabolism, characterized by a reduction in OXPHOS and activation of alternative energy production pathways. ONC206 treatment in vivo at well-tolerated doses produces significant tumor growth inhibition and improves overall survival in two independent UM PDX models. Collectively, these findings suggest that ONC206 represents a promising therapeutic candidate warranting further clinical evaluation in patients with mUM. 

## 2. Materials and Methods

### 2.1. Antibodies and Reagents

Antibodies against SDHA (ab14715) and LC3B (ab48394) were purchased from Abcam (Cambridge, UK), and the cleaved PARP antibody (#5625s) was obtained from Cell Signaling Technology (Danvers, MA, USA). Antibodies for β1-actin (sc47778) and SDHB (sc271548) were purchased from Santa Cruz Biotechnology (Dallas, TX, USA). ONC206 was supplied by Oncoceutics/Chimerix (Durham, NC, USA) under a material transfer agreement. ONC206 was dissolved in water. Accordingly, water was used as a vehicle control in all in vivo assays.

LentiBrite™ RFP-LC3 Lentiviral Biosensor was obtained from Sigma-Aldrich, St Louis, MO, USA (Cat. No. 17-10143) and Acridine orange from Invitrogen, Waltham, MA, USA (#A3568).

### 2.2. Cell Lines

UM cells were cultured in RPMI 1640 media with 10% FBS, 1% glutamine, 1% penicillin–streptomycin, 1% HEPES, and 1% insulin supplement, under ambient oxygen at 37 °C. Cell line MEL20-06-039 (primary cell line; RRID: CVCL_8473) [18,19] was obtained from Dr. Tara A. McCannel. Cell lines OMM1 (metastatic cell line; RRID: CVCL_6939) [20], OMM2.3 (metastatic cell line; RRID: CVCL_C306) [20], OMM2.5 (metastatic cell line; RRID:CVCL_C307) [21], and MEL270 (primary cell line; RRID: CVCL_C302) were provided by Drs. Martine Jager and Bruce Ksander. MM28 (metastatic cell line) was obtained from ATCC. Additional UM cell line information is provided in the references [21,22,23,24].

Cell lines were validated by short random repeat (STR) DNA fingerprinting techniques and mutational analysis, by the MD Anderson Cancer Center Support Grant-supported Characterized Cell Line Core, using the AmpFLSTR Identifier Kit (Applied Biosystems, Foster City, CA, USA). The STR profiles were compared to known ATCC fingerprints (ATCC.org), and to the Cell Line Integrated Molecular Authentication database version 0.1.200808 (http://bioinformatics.istge.it/clima/; accessed on 1 June 2025). The STR profiles matched known DNA fingerprints or were unique. Cell cultures were checked for Mycoplasma contamination every 6 months using the MD Anderson Cancer Center Cytogenetic and Cell Line Authentication Core.

### 2.3. Western Blotting

Cells were lysed in a buffer containing 50 mM Tris (pH 7.9), 150 mM NaCl, 1% NP40, 1 mM EDTA, 10% glycerol, 1 mM sodium vanadate, and a protease inhibitor cocktail (Roche Pharmaceuticals, Nutley, NJ, USA). Proteins were separated by SDS-PAGE with 14% gradient gels (Invitrogen, Waltham, MA, USA), transferred to a Hybond-ECL nitrocellulose membrane (Cytiva, Wilmington, DE, USA) and blocked in 5% dry milk in PBST. The membrane was then incubated with primary and secondary antibodies, and target proteins were detected with ECL detection reagent (Thermo Fisher Scientific, Waltham, MA, USA). Antibodies used are described in Section 2.1.

### 2.4. Colony Formation Assay

UM cells were seeded in 24-well plates at 300 cells/well. Five days later, colonies were formed, and ONC206 (0.1, 0.25, 0.5, and 1.0 μM) was added to the wells. Controls were untreated cells. Treatment was continued and colonies were allowed to grow until they covered 70–80% of the well surface in the control wells. For fixing and staining the cells, the culture media was removed, wells were washed with 1X PBS, and 1 mL of crystal violet in 25% methanol was added. Cells were stained for 5 min at RT. Crystal violet was aspirated, and the wells were washed with water until only colony staining remained.

### 2.5. Annexin V and Propidium Iodide Flow Cytometry

Cells were plated in 10 cm plates at 5.0 × 10^5^ density, cultured overnight, and treated with ONC206 (0.25 and 0.5 μM) for 72 h. Controls were untreated cells. At the time of collection, supernatants were collected, and adherent cells were trypsinized for 5–10 min. Trypsinized cells were combined with the corresponding supernatant. Cells were pelleted by centrifugation at 500 *g* for 2.5 min, resuspended in Annexin V-binding buffer (ABB), counted, and diluted in ABB to 1.0 × 10^6^ cells/mL. Of this, 100 μL of the cells was aliquoted into 1 mL tubes and stained by adding 1 μL of 100 μg/mL propidium iodide and 5 μL of Annexin V FITC for flow cytometry. Samples were measured at a fluorescence emission of 530 nm.

### 2.6. Autophagy Assay

#### 2.6.1. Acridine Orange Staining and Confocal Microscopy

Live-cell imaging was performed on UM cells using an Andor spinning disk confocal microscope (Andor, Belfast, UK) to evaluate autophagic activity. Cells were seeded in ibidi-treated 8-well polymer-bottom plates at a density of 5000 cells/well. The next day, cells were treated with ONC206 (0.5 μM) and rapamycin (1.0 μM) as the positive control. Treatment lasted for 48 h. Chloroquine-treated cells were used as a negative control (1 μM, 24 h). For cell staining, Acridine orange (1 mg/mL) diluted in phenol red-free culture media to a final concentration of 1 μg/mL was added and incubated for 10 min in the dark, washed gently with PBS, and maintained in phenol red-free media for imaging. Confocal microscopy was performed with an Andor WD spinning disk confocal microscope utilizing an iXon Ultra EMCCD Camera (Oxford Instruments Andor, Belfast, UK) and a 20x 0.70 NA objective. Fluorescence was captured with a 488 nm excitation laser for green fluorescence (emission collected at 525 nm) and a 561 nm laser for red fluorescence (emission collected at 620 nm). Identical acquisition parameters were used for all conditions.

#### 2.6.2. RFP-LC3 Lentiviral Biosensor Assay and Live-Cell Confocal Microscopy

Live-cell imaging of autophagic flux was performed in UM cells MM28 and MEL20-06-039 using the LentiBrite™ RFP-LC3 Lentiviral Biosensor. Cells were seeded at 5000 cells/well in ibiTreat μ-Slide 8-well polymer-bottom plates (ibidi) and allowed to adhere for 24 h. ONC206, rapamycin and chloroquine treatments were done as mentioned before. To avoid dilution of the lentiviral biosensor signal, lentiviral infection was carried out after drug addition and timed to precede imaging by approximately 16–18 h. Cells were transduced with the RFP-LC3 lentivirus at 10 multiplicities of infection in 10% FBS phenol red-free media. The next morning, lentivirus was removed, and cells were washed with PBS and maintained in the above media. Live-cell confocal imaging was performed on the Andor spinning disk confocal microscope using a 561 nm excitation laser and a 620 nm emission filter to detect RFP fluorescence and pics were taken utilizing an iXon Ultra EMCCD Camera.

### 2.7. Reverse Phase Protein Array (RPPA) Analysis

RPPA analyses were performed at the UT MD Anderson Cancer Center’s Functional Proteomics RPPA Core. Cell lysates were two-fold serially diluted over five dilutions (undiluted to 1:16) and arrayed on nitrocellulose-coated slides. Samples were probed with antibodies using catalyzed signal amplification and visualized by 3,3′-diaminobenzidine colorimetric reaction. Slides were scanned to produce 16-bit TIFF images, and spot densities were quantified using the MicroVigene software (V 3.0, accessed on 1 Decemebr 2024) program. Relative protein levels were determined by interpolation of each dilution curve from the “standard curve” generated using an R script written by MD Anderson’s Department of Bioinformatics & Computational Biology. Heatmaps were generated in Cluster 3.0 (http://www.eisenlab.org/eisen/; accessed on 1 January 2025) as a hierarchical cluster using Pearson’s correlation and a center metric.

### 2.8. Cell Viability Assays

Methylthiazole tetrazolium (MTT; 3-(4,5-dimethylthiazol-2-yl)-2,5-diphenyltetrazolium bromide)-based assays were used for estimating cell viability. UM cells were plated at a density of 3 × 10^4^ cells/well in triplicate in a 24-well plate. MTT reagent (Sigma-Aldrich, St. Louis, MO, USA) was dissolved in PBS to a final concentration of 1 mg/mL, and 200 μL was added to each well. After 2 h, the precipitate formed was dissolved in DMSO, and the color intensity was estimated in an MRX Revelation microplate absorbance reader (Dynex Technologies, Chantilly, VA, USA) at 570 nm.

### 2.9. Transfection with siRNA

MM28 and MEL20-06-039 cells were plated at a density of 3 × 10^4^ cells/well in duplicate in a 24-well plate. Cells were then transfected with siRNA targeting CLPP (siCLPP) or a nontargeting (siNT) control. Both siRNAs were used at a final concentration of 10 nM using Lipofectamine according to the manufacturer’s protocol. After 6 h, the transfection mixture was removed, and the cells were maintained in fresh culture medium overnight. The cells were then treated with ONC206 (0.25 and 0.5 μm) for 72 h, while control cells remained untreated. Methylthiazole tetrazolium (MTT)-based cell viability assays were used to estimate cell survival, as described in the method mentioned above.

### 2.10. Seahorse Mito Stress Test

Mitochondrial respiration was assessed using the Seahorse XF Cell Mito Stress Test Kit (Agilent, #103015-100; Santa Clara, CA, USA) on a Seahorse XFe96 Analyzer following the manufacturer’s instructions. Cells (1.5 × 10^4^ per well) were seeded in Seahorse XF96 plates, allowed to adhere overnight, and treated with the indicated concentrations of ONC206 for 8 h. Prior to the assay, culture medium was replaced with Seahorse XF DMEM assay medium supplemented with 10 mM glucose, 2 mM L-glutamine, and 1 mM sodium pyruvate, followed by incubation for 45 min at 37 °C in a non-CO_2_ incubator. Oligomycin (1 μM), FCCP (0.5 μM), and rotenone/antimycin A (0.5 μM) were sequentially injected to measure oxygen consumption rate (OCR). After the assay, nuclei were stained with Hoechst 33342, imaged using a Cytation 5 system, and cell numbers were used for normalization. Mitochondrial respiration parameters were calculated from OCR data using Wave software (V 2.6) and normalized to cell number.

### 2.11. Metabolomic Profile Analysis

Metabolites were extracted using ice-cold 80/20 (*v*/*v*) methanol/water with 0.1% ammonium hydroxide. Extracts were centrifuged at 17,000× *g* for 5 min at 4 °C, and supernatants were transferred to clean tubes, dried under nitrogen, reconstituted in deionized water, and analyzed by ion chromatography (IC)-HRMS using a Thermo Scientific Dionex ICS-6000+ system with an IonPac AS11 column. Mobile phases consisted of water and 100 mM KOH with a flow rate of 360 μL/min, with a run time of 55 min. Data were acquired using a Thermo Orbitrap IQ-X Tribrid Mass Spectrometer (Thermo Fisher Scientific, Waltham, MA, USA) under ESI negative ionization mode. The relative abundance of each metabolite was normalized by total area, log-transformed and scaled by z-score, and metabolic pathways were analyzed using R scripts written in-house.

### 2.12. Lipidomic Profile Analysis

To each cell sample, 200 μL of extraction solution containing 2% Avanti SPLASH^®^ LIPIDOMIX^®^ Mass Spec Standard and 1% 10 mM butylated hydroxytoluene in ethanol was added and vortexed for 10 min, incubated on ice for 10 min and centrifuged at 13,300× *g* for 10 min at 4 °C. Ten microliters of supernatant was injected for analysis. Mobile phases were acetonitrile/water with 0.1% formic acid and 10 mM ammonium formate, and isopropanol/acetonitrile with a flow rate of 0.200 mL/min. A Thermo Fisher Scientific Orbitrap Fusion Lumos Tribrid mass spectrometer was operated in data-dependent acquisition mode, with scan ranges of 150–827 and 825–1500 *m*/*z*. An Orbitrap resolution of 120,000 (FWHM) was used for MS1 acquisition and a spray voltage of 3600 and −2900 V were used for positive and negative ionization modes, respectively. Lipid data were processed and annotated using Thermo Fisher Scientific LipidSearch software (version 5.0) and analyzed using R scripts written in-house.

### 2.13. UM PDX Models

To generate the UM PDX model 003.217, informed consent was obtained from the patient for collecting liver biopsy tissue under the IRB-approved protocol (Dr. Meric-Bernstam). This patient received prior treatment with ipilimumab and nivolumab. Tumor fragments from image-guided liver biopsies were implanted on the dorsal flank of NOD SCID gamma (NSG) mice (Jackson Labs, Bar Harbor, Maine, USA). Mice were anesthetized in an isoflurane chamber. A small incision (~5 mm) was made to create a pocket under the skin. Then, a tumor piece (~2–3 mm in diameter) was placed into the pocket, and the wound was sealed with a wound clip. Each mouse was individually tagged to follow-up for individual tumor growth. Tumor measurements were performed using calipers, and tumor volume (TV) was calculated using the formula TV (mm^3^) = (width^2^ × length)/2. Tumor volume and body weight were assessed twice a week. When tumors reached >2000 mm^3^ volume, tumor tissues were collected and transferred into new NSG mice for the next passage. Tumor samples were also stored in FFPE as well as snap-frozen for potential future molecular analysis. Tissues from the first passage were used for genetic characterization, revealing that this was a GNA11 mutant (Q209L) UM.

The UM PDX model WM4481-1 was generously provided by Dr. Meenhard Herlyn’s group at The Wistar Institute, Philadelphia, PA, USA. This model was derived from a liver metastasis of uveal melanoma from a patient previously treated with pembrolizumab and temozolomide. Molecular profiling of the model demonstrated the presence of GNA11 (Q209L) and SF3B1 (R625C) mutations.

### 2.14. In Vivo Treatment of UM PDX

All in vivo studies were performed in accordance with the accepted guidelines for housing, euthanasia, and treatment under an Institutional Animal Care and Use Committee (IACUC)-approved protocol at MD Anderson. Flank tumors were established as described above and grown to a size of ~75–150 mm^3^. The mice (*n* = 8 per group) were then randomized to receive treatment with either vehicle (water) or ONC206 (50 mg/kg). ONC206 was administered via oral gavage, twice a day for three consecutive days every week until the end of the experiment. ONC206 dose and administration frequency were based on information provided by Jazz Pharmaceuticals. Tumor measurements were performed twice weekly using slide calipers by blinded observers. Tumors were excised at 6 weeks during treatment and formalin-fixed for immunohistochemistry and snap-frozen for molecular analyses.

## 3. Results

### 3.1. ONC206 Inhibits Oxphos and Cell Growth in UM Cells In Vitro

**Figure 1:**
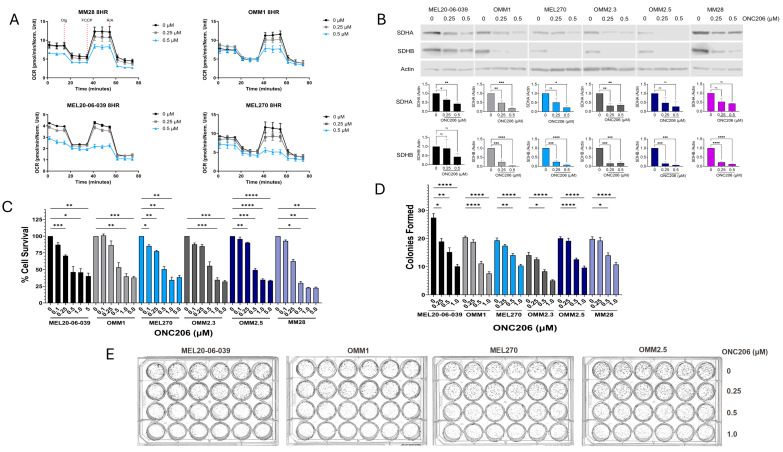
ONC206 inhibits OXPHOS and cell survival in UM cells. (**A**) Analysis of mitochondrial respiration after 8 h of ONC206 treatment (0.25 and 0.5 μM) using Mito Stress Test Seahorse assay in UM cells (MM28, OMM1, MEL20-06-039, MEL270). The arrows represent the time points of addition of Oligomycin, FCCP, and rotenone/antimycin (R/A). The Y-axis represents oxygen consumption rate (OCR, pmol/min) normalized by cell number in corresponding sample well. (**B**) Western blot analysis (upper panel) of UM cell lines (primary cell lines [MEL20-06-039, MEL270], and metastatic cell lines [OMM1, OMM2.3, OMM2.5, and MM28]) treated with ONC206 for 48 h to detect SDHA and SDHB (* *p* < 0.05; ** *p* < 0.01; *** *p* < 0.001; **** *p* < 0.0001). Lower panel shows quantitation of SDHA and SDHB expression levels from the Western blots; actin was used for normalization. (**C**) Effect of ONC206 (0.1, 0.25, 0.5, 1.0, and 5.0 μM) on UM cell survival in MEL20-06-039, OMM1, MEL270, OMM2.3, OMM2.5, and MM28 cell lines by MTT-based cell survival assay; bar graphs represent mean ± SEM of three independent experiments, *p*-values were calculated by comparison to untreated controls (* *p* < 0.05; ** *p* < 0.01; *** *p* < 0.001; **** *p* < 0.0001). (**D**) Colony formation assay: The average number of colonies observed after staining with crystal violet in ONC206 treated (0.1, 0.25, 0.5, and 1.0 μM) and untreated control UM cell lines (MEL20-06-039, OMM1, MEL270, OMM2.3, OMM2.5, and MM28) as demonstrated in (**E**), was plotted. A total of 5 fields/condition/cell line were counted to obtain the average number of colonies. The bar graphs represent average number of colonies per field and are the mean ± SEM of three independent experiments. *p*-values were calculated by comparing untreated controls with ONC206 treatment doses (* *p* < 0.05; ** *p* < 0.01; **** *p* < 0.0001).

ONC206 treatment significantly inhibited mitochondrial basal, maximal, and spare capacity respiration in UM cells to variable degrees, as reflected by the normalized oxygen consumption rate (OCR) (Figure 1A) in a Mito Stress Test assay. ONC206 treatment also reduced the levels of SDHA and SDHB, which are OXPHOS effector proteins, in the UM primary cell lines MEL20-06-039 and MEL270, and metastatic cell lines OMM1, OMM2.3, OMM2.5, and MM28, as detected by Western blot analysis (Figure 1B, Figures S1A, S8 and S9). This effect is mediated by activation of CLPP mitochondrial protease [17] (Figure S1B). MTT assays showed dose-dependent reduction in cell viability of UM cells by ONC206 treatment (Figure S1C). As the MTT assay relies on cellular redox activity and thus could potentially reflect indirect effects of ONC206 on redox metabolism, we also tested the effect of ONC206 treatment in a clonogenic colony formation assay. This again showed that ONC206 significantly inhibited colony formation by the UM cell lines (Figure 1D,E and Figure S1C), confirming its robust antiproliferative effect.

### 3.2. Treatment with ONC206 Induces Autophagy

**Figure 2:**
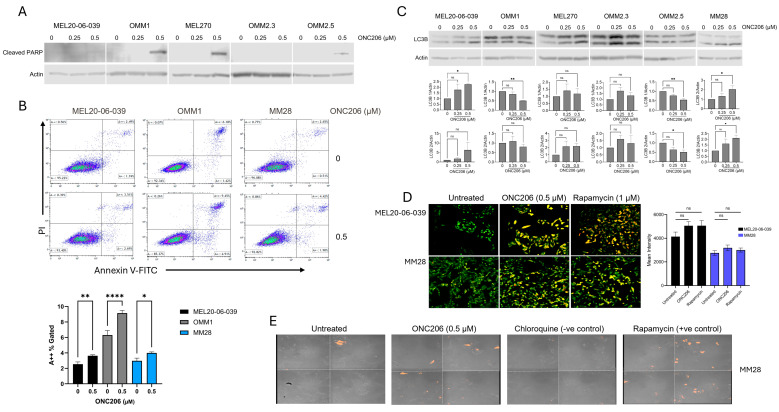
ONC206 differentially induces apoptosis or autophagy in UM. (**A**) Western blot analysis of cleaved PARP following 72 h treatment with ONC206 (0.25 and 0.5 μM). (**B**) Annexin staining of ONC206-treated (0.5 μM) MM28 (metastatic), MEL20-06-039 (primary) and OMM1 (metastatic) cell lines for detection of apoptotic cells. Upper panel shows histogram of annexin-positive cell distribution and lower panel shows bar graph of percent annexin-positive cells (* *p* < 0.05; ** *p* < 0.01; **** *p* < 0.0001). (**C**) Detection of autophagy marker LC3B levels (upper panel) in UM cells (MEL20-06-039, primary; OMM1, metastatic; MEL270, primary; OMM2.3, metastatic; OMM2.5, metastatic; and MM28, metastatic) using Western blot analysis after treatment with ONC206 (0.25 and 0.5 μM). Lower panel shows quantitation of LC3B levels (* *p* < 0.05; ** *p* < 0.01). (**D**) Imaris rendering of confocal microscopic images and quantitation of Acridine orange staining of UM cells (MEL20-06-039 and MM28) for detection of autophagic vesicles after treatment with ONC206 (0.5 μM); rapamycin treatment is used as positive control. (**E**) Imaris rendering of confocal microscopic images of lentivirally LC3B-RFP (red fluorescent protein)-infected UM cell MM28 treated with 0.5 μM ONC206 for detection of autophagy marker LC3B. Rapamycin and chloroquine treatment serve as positive and negative controls.

We performed apoptosis and autophagy assays to further investigate the growth inhibitory effect of ONC206 in UM. MEL270 (primary) and OMM1 and OMM2.5 (metastatic) cell lines demonstrated PARP cleavage (Figure 2A, Figures S2 and S10), consistent with apoptosis induction with ONC206 treatment. However, this was not seen in MEL20-06-039 (primary) and OMM2.3 (metastatic) cell lines and was minimally observed in the MM28 (metastatic) cell line. With a more sensitive flow cytometry-based annexin assay, induction of apoptosis was detected in MEL20-06-039, OMM1 and MM28 cell lines (Figure 2B and Figure S10) with ONC206 treatment. We also evaluated the UM cells for evidence of autophagy following ONC206 treatment. MEL20-06-039, MEL270, OMM2.3, and MM28 cells demonstrated increased expression of the autophagy marker LC3B with ONC206 (Figure 2C). The induction of autophagy was confirmed by Acridine orange staining of autophagosomes in UM cell lines MM28 and MEL20-06-039 (Figure 2D). These findings were further corroborated by lentiviral transduction of the UM cell line MEL20-06-039 with LC3B–red fluorescent protein (RFP), enabling the visualization of increased LC3B levels following ONC206 treatment (Figure 2E). Chloroquine and rapamycin were employed as negative and positive controls, respectively, for autophagy induction.

### 3.3. UM Cells MM28 Demonstrate Altered Metabolic Profiles After ONC206 Treatment

**Figure 3:**
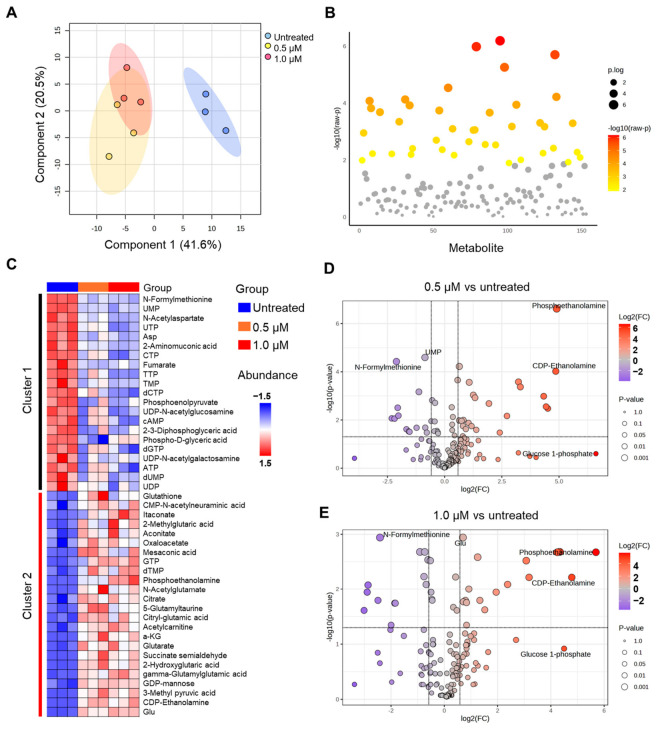
ONC206 treatment for 24 h induces dose-dependent metabolic reprogramming in MM28 (metastatic cell line) through OXPHOS pathway disruption. (**A**) Principal component analysis showing distinct metabolic clustering of untreated versus ONC206-treated (0.5 and 1.0 μM) MM28 cell samples in a dose-dependent manner. (**B**) ANOVA volcano plot identifying 42 significantly altered metabolites following ONC206 treatment with statistical significance and effect sizes. (**C**) Hierarchical clustering heatmap of significantly altered metabolites revealing two distinct clusters with opposing dose-dependent response patterns. (**D**) Volcano plot comparing 0.5 μM ONC206 versus untreated samples showing fold-change and statistical significance of metabolite alterations. (**E**) Volcano plot comparing 1.0 μM ONC206-treated versus untreated samples demonstrating enhanced metabolic effects at higher drug concentrations.

Imipridones impact mitochondrial metabolism, and our previous studies show a significant alteration in the metabolic landscape of UM cells following treatment with imipridone compounds [17]. Therefore, we performed global metabolic profiling of MM28 (metastatic cell line), which represents poor-prognosis monosomy 3 uveal melanoma. After treatment of MM28 cells with 0.5 μM and 1.0 μM ONC206 for 24 h, we performed HILIC and then a semi-targeted approach for comprehensive metabolomic profiling using ion chromatography–mass spectrometry (IC-MS). Principal component analysis (PCA) of the metabolomic data revealed distinct metabolic signatures between treated and untreated UM cells (Figure 3A). Untreated control samples clustered tightly, indicating consistent baseline metabolic profiles. In contrast, ONC206-treated samples demonstrated dose-dependent metabolic reprogramming, with 0.5 μM treated MM28 samples forming an intermediate cluster and 1.0 μM treated samples demonstrating the most pronounced metabolic alterations. These results further underscore the specificity of drug action. The clear separation between the two treatment groups along Component 1 suggests that ONC206 induces systematic changes in cellular metabolism in a dose-dependent manner. ANOVA analysis identified 42 metabolites that were significantly altered across treatment conditions (*p* < 0.05) (Figure 3B). The distribution of significant metabolites (colored points) versus non-significant ones (gray points) illustrates that while ONC206 treatment in MM28 cells causes selective metabolic changes, the majority of detected metabolites remain unchanged, suggesting targeted rather than global metabolic disruption (Figure 3B). Two distinct metabolic clusters were identified (Figure 3C). Cluster 1 consists of metabolites that showed dose-dependent decreases with ONC206 treatment. This cluster is enriched with key OXPHOS-related metabolites including nucleotide phosphates such as UMP, UTP, CTP, TTP, ATP, dGTP, dCTP, and dUMP, which are essential for energy metabolism and directly dependent on mitochondrial ATP synthesis. Cluster 1 also includes glycolytic intermediates like phosphoenolpyruvate, 2-3-diphosphoglyceric acid, and phospho-D-glyceric acid, likely due to compensatory upregulation of glycolysis when OXPHOS is not functional, as well as TCA cycle components such as fumarate that are directly linked to Complex II (SDHA/SDHB) activity.

Cluster 2 consisted of metabolites that increased with ONC206 treatment. This cluster includes amino acid derivatives and organic acids such as glutathione, itaconate, citrate, α-ketoglutarate, and glutarate, suggesting metabolic stress responses and activation of alternative energy production pathways (Figure 3C). The increase in lipid metabolism intermediates like acetylcarnitine indicates shifts toward fatty acid oxidation when OXPHOS is compromised. This dose-dependent metabolic reprogramming reflects SDHA/SDHB inhibition, as demonstrated by the correlation between decreasing SDHA and SDHB protein levels with increasing ONC206 concentrations (Figure 1B and Figure S1A) and the progressive impairment of Complex II activity. The metabolic disruption is characterized by reduced ATP production, indicated by lower nucleotide phosphate levels, disruption of the TCA cycle with altered intermediates such as fumarate and citrate, and activation of compensatory stress responses, evidenced by increased glutathione and other stress-related metabolites. Differentially altered metabolite analysis using a volcano plot also suggests significant modulation of OXPHOS-associated metabolites with ONC206 treatment (Figure 3D,E). All significant metabolite changes induced by ONC206 are presented as a heat map in Figure S3, while redox, glycolysis, and nucleotide metabolism-related changes are represented in separate heat maps (Figure S4).

### 3.4. UM Cells MM28 Display Reprogrammed Global Lipid Metabolism After ONC206 Treatment

**Figure 4:**
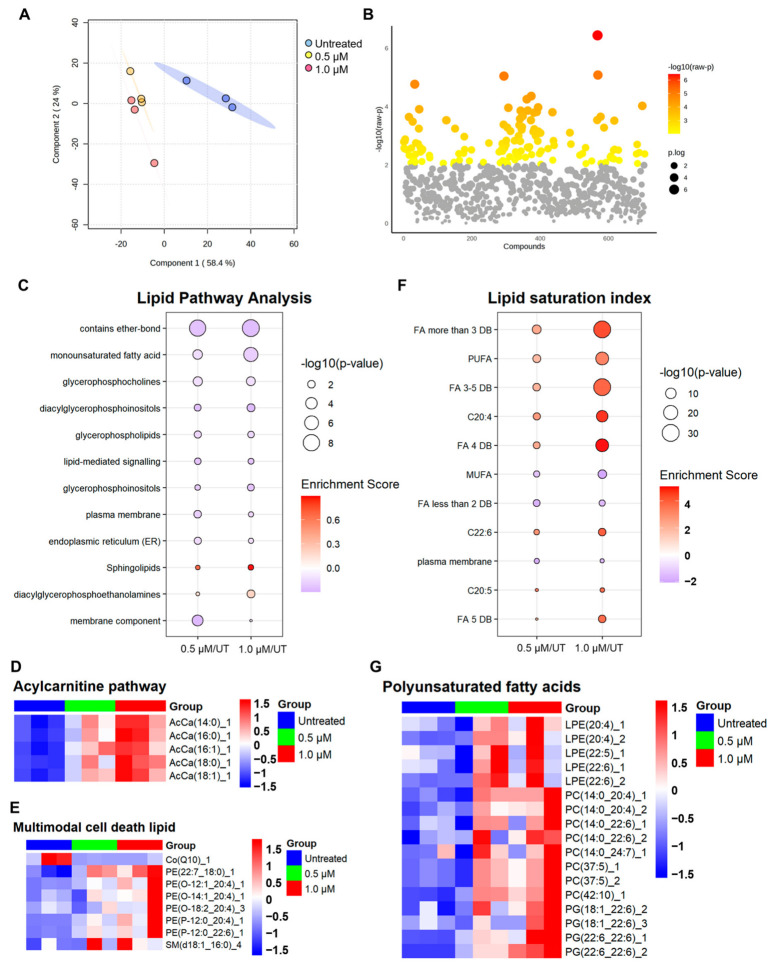
Alterations in lipid profile of ONC206-treated (24 h) MM28 (metastatic cell line). (**A**) Principal component analysis showing distinct clustering of lipid profiles in untreated versus ONC206-treated (0.5 and 1.0 μM) MM28 cell samples in a dose-dependent manner. (**B**) ANOVA volcano plot identifying significantly altered lipids following ONC206 treatment with statistical significance and effect sizes. (**C**) Lipid pathway analysis showing alterations in different classes of lipids in MM28 cells based on biological function or type after ONC206 treatment with statistical significance and effect sizes. (**D**,**E**) Alterations in acetyl carnitine pathway and cell death lipid components after treatment with ONC206. (**F**) Changes in lipid saturation post-treatment with ONC206 with statistical significance and effect sizes. (**G**) Dose-dependent increase in polyunsaturated fatty acids due to ONC206 treatment.

To comprehensively understand the metabolic impact of ONC206 treatment on UM cells and since not all metabolites and lipids are extractable with a single buffer (aqueous or alcohol-based), we performed a separate global lipidomics analysis on MM28 (metastatic cell line) treated with 0.5 μM and 1.0 μM ONC206 for 24 h. Principal component analysis (PCA) revealed distinct clustering of treated samples from untreated controls (Figure 4A), with Component 1 (58.4%) and Component 2 (24.9%) capturing 83.3% of the total variance, indicating substantial lipid metabolic reprogramming. The volcano plot analysis (Figure 4B) identified over 100 significantly altered lipid species (*p* < 0.05, |log2(FC)| > 1), with the most dramatic changes observed in phospholipids, acylcarnitines, and polyunsaturated fatty acids, consistent with our other metabolomics studies indicating disrupted energy metabolism and increased oxidative stress.

Pathway enrichment analysis (Figure 4C) revealed dose-dependent alterations in multiple lipid metabolic pathways. Overall, the analysis indicates a dose-dependent effect of ONC206, with the most prominent changes observed in sphingolipids and diacylglycerophosphoethanolamines, which show higher enrichment at 1.0 μM treatment. In contrast, membrane lipids and fatty acid metabolic pathways exhibited decreased enrichment as the ONC206 dose increased. Notably, pathways associated with ether-bond lipids, monounsaturated fatty acids, and diacylglycerophosphoethanolamines exhibit progressive activation with increasing drug concentration (*p* < 0.01), suggesting dose-dependent membrane remodeling and altered lipid signaling cascades.

The acylcarnitine pathway analysis (Figure 4D) demonstrated a striking pattern of metabolic rewiring that correlated directly with our earlier observations of disrupted mitochondrial function and TCA cycle intermediates. Short- and medium-chain acylcarnitines [AcCa(14:0), AcCa(16:0), AcCa(16:1), AcCa(18:0), and AcCa(18:1)] exhibited dose-dependent accumulation in treated cells, with log2(FC) values ranging from −1.0 in untreated cells to +1.4 in 1.0 μM treated cells. This accumulation pattern indicates incomplete fatty acid β-oxidation, corroborating our metabolomics data showing elevated N-Acetylcarnitine (AcCa) and succinate levels, and supporting our Western blot findings of decreased mitochondrial Complex I and II expression. The multimodal cell death lipid profile (Figure 4E) further substantiated these findings, revealing significant elevation of lipid species associated with both apoptosis and autophagy, including PE(22:7_18:0), PE(O-14:1_20:4), PE(P-12:0_20:4), and SM(d18:1_16:0). These changes occurred predominantly at the 1.0 μM dose, suggesting that sustained lipid oxidation triggers multiple cell death pathways.

Remarkably, the lipid saturation index analysis (Figure 4F,G) uncovered a unique metabolic adaptation in ONC206-treated cells. While most fatty acid species showed expected dose-dependent changes, polyunsaturated fatty acids (PUFAs) exhibited a striking accumulation pattern. Long-chain PUFAs, including LPE(20:4), LPE(22:5), LPE(22:6), PC(14:0_20:4), PC(14:0_22:6), PC(37:5), PC(42:10), PG(18:1_22:6), and PG(22:6_22:6) (Figure 4G), showed significantly elevated levels in both 0.5 μM and 1.0 μM treated groups compared to untreated controls (*p* < 0.01, log2(FC) > 0.5). This PUFA enrichment was particularly evident in phosphatidylcholine (PC) and phosphatidylglycerol (PG) species containing arachidonic acid (20:4) and docosahexaenoic acid (22:6). Given that PUFAs are highly susceptible to oxidative damage and their oxidation products are potent inducers of lipid peroxidation-mediated cell death, this PUFA accumulation likely represents a critical vulnerability induced by ONC206. Indeed, the PUFA enrichment pattern directly aligns with our metabolomics observations of increased oxidative stress markers and decreased glutathione levels, suggesting that ONC206 sensitizes UM cells to oxidative death by simultaneously increasing PUFA content while depleting antioxidant defenses. A comprehensive list of global lipid analysis results is provided in Figure S5. Together, these comprehensive lipidomics data reveal that ONC206 induces coordinated reprogramming of lipid metabolism, characterized by incomplete fatty acid oxidation, membrane remodeling, and PUFA accumulation, ultimately converging on oxidative stress-mediated cell death pathways.

### 3.5. ONC206 Inhibits Tumor Growth and Improves Survival in UM PDX Models

**Figure 5:**
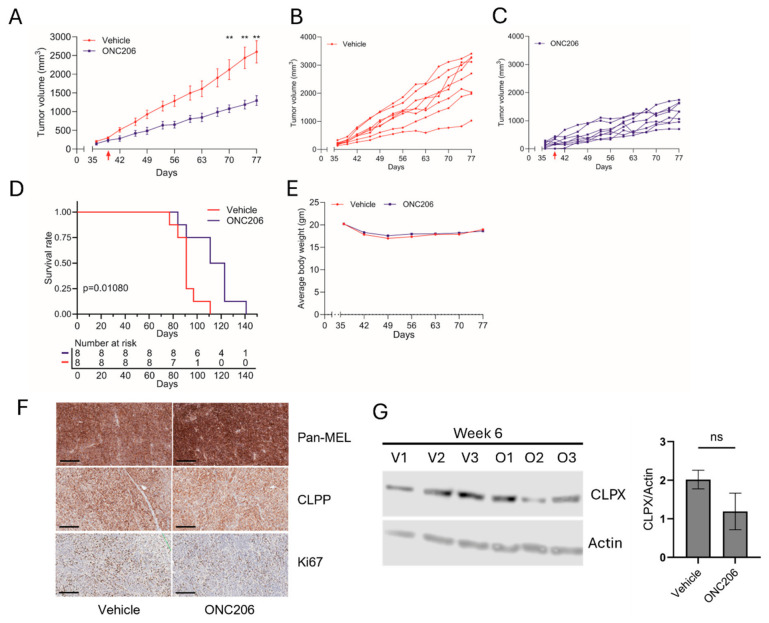
ONC206 treatment reduces tumor burden and improves survival of UM PDX.003.217 model in mice. (**A**) Tumor growth curves plotted from tumor volume measurements 2× weekly post-tumor initiation (** *p* < 0.01). The red arrow indicates the beginning of ONC206 treatment. (**B**,**C**) Individual mouse tumor growth plots in vehicle and ONC206-treated groups. The red arrow indicates the beginning of ONC206 treatment (**D**) Kaplan–Meier plots of UM PDX.003.217 model with ONC206 treatment compared to vehicle-treated controls; *n* = 8 per treatment group; vehicle vs. ONC206, *p* = 0.01080. (**E**) Mouse body weight curves with vehicle and ONC206 treatment. (**F**) Representative images (scale bar = 100 μm) of pan-melanoma, CLPP, and Ki67 immunohistochemical staining in vehicle and ONC206-treated tumors. (**G**) Western blot analysis and quantitation of ONC206 treatment biomarker CLPX in three vehicle-treated (V1–V3) and three ONC206-treated (O1–O3) mice tumors at week 6.

UM PDX.003.217 model was used to investigate the effect of ONC206 treatment. Tumor growth to approximately 100 mm^3^ was confirmed before mice were randomized to receive either 50 mg/kg ONC206 or vehicle (water) twice daily for three consecutive days each week. Tumor volume was monitored twice weekly. ONC206 treatment significantly reduced tumor volume (Figure 5A–C) over time and improved survival compared to vehicle-treated mice (Figure 5D) (*p* = 0.0108, Cox HR = 5.021 (95% CI: 1.361–18.522). Median survival of vehicle- and ONC206-treated groups was 91 and 117 days, respectively. ONC206 was well-tolerated, and no significant difference in the body weights of the mice was observed over the course of the experiment (Figure 5E). A significant reduction in tumor size and improved survival with ONC206 treatment was confirmed in a second UM PDX model, WM4481-1 (Figures S6 and S11).

### 3.6. ONC206 Alters Proteomic Profiles of UM

PDX.003.217 tumor tissue was collected 6 weeks after the start of treatment for characterization of the in vivo effects of ONC206. IHC analysis of the tumors from the 6-week time point displayed strong pan-melanoma and CLPP marker staining in all samples, with a reduction in Ki67 staining in ONC206-treated tumors (Figure 5F and Figure S7). Western blot analysis of the tumors from ONC206- and vehicle-treated mice demonstrated downregulation of CLPX (biomarker for ONC206 activity) (Figure 5G and Figure S12).

**Figure 6:**
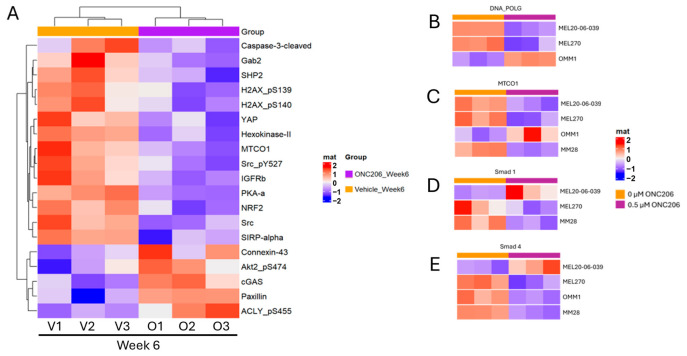
ONC206 treatment of UM PDX.003.217 and UM cells alters their proteomic profile. (**A**) Heatmap showing proteomic alterations in mice tumors at 6 weeks of treatment with ONC206; V1–V3 and O1–O3 are three replicates of vehicle-treated (yellow bar) and ONC206-treated (purple bar) mice tumors, respectively, after RPPA analysis. (**B**–**E**) Heatmaps showing significant proteomic changes observed in primary UM cell lines MEL20-06-039, MEL270, and metastatic cell lines OMM1 and MM28 after ONC206 treatment for 8 h or 24 h at a 0.5 μM dose followed by RPPA analysis. Untreated cells (yellow bars) were compared with the ONC206-treated cells (purple bars).

In order to more broadly characterize the effects of ONC206 on proteins, phosphoproteins, and protein signaling networks, we also performed RPPA analysis on the PDX.003.217 tumor tissue collected after 6 weeks of treatment (Figure 6). Differential protein expression between the treatment groups was analyzed using one-way ANOVA, and candidates were selected based on statistical significance (*p* < 0.05) and a fold change > 1.5, as shown in the heatmap. The proteomic profile (Figure 6A) indicates that ONC206 profoundly remodels several interconnected survival pathways in tumor cells. It markedly suppresses receptor tyrosine kinase signaling, as evidenced by reduced SHP2, IGF-1 receptor β, total Src, inhibitory Src_pY527 [25], phosphorylated YAP [26], and PKA-α.

In parallel, ONC206 treatment resulted in lower levels of MTCO1 and NRF2 proteins, consistent with impaired mitochondrial ATP production and a reduced capacity to buffer reactive oxygen species. At this time point, cleaved caspase-3 is lower in ONC206-treated tumors than in vehicle controls, likely implying that an earlier wave of caspase-dependent apoptosis has subsided and that residual cells are now growth-arrested or diverted toward non-caspase-mediated death pathways such as autophagy. ONC206 strongly induced the cytosolic DNA sensor cGAS while reducing expression of the myeloid checkpoint receptor, SIRP-α, suggesting leakage of damaged nuclear or mitochondrial DNA into the cytosol and activation of the cGAS–STING/type I interferon axis. Collectively, these pathway-level alterations support a model in which ONC206 eliminates tumor cells by shutting down receptor tyrosine kinase/Src/YAP-driven growth [27] and mitochondrial–redox homeostasis, and by shifting residual cells into a metabolically fragile state.

ONC206-treated tumors also show increased phosphorylation of Akt2 on S474 [28], activation of the lipogenic enzyme ACLY, and upregulation of paxillin and connexin 43, consistent with adaptive rewiring of surviving cells toward Akt-dependent survival, altered lipid metabolism, and enhanced focal adhesion and gap junction–mediated cell–cell communication. These changes may transiently support persistence of resistant subpopulations, which may eventually lead to overall treatment resistance or disease relapse.

We also observed similar changes in the proteomics analysis of UM cells treated in vitro with ONC206. RPPA analysis of UM primary cell lines MEL270 and MEL20-06-039, and metastatic cell lines MM28 and OMM1 treated with 0.5 μM of ONC206 for 8 h and 24 h showed alterations in proteins associated with mitochondrial function and TGF-β signaling. Across models, ONC206 downregulated DNA POLG (sole mitochondrial polymerase) and MTCO1 (complex IV crucial for ATP generation) at the 24 h time point (not significantly altered at the 8 h time point), indicating impaired mitochondrial maintenance (Figure 6B,C). Additionally, we noted changes in the TGF-β signaling (Smad 1 at 8 h and Smad 4 at 24 h) in the cells treated with ONC206 (Figure 6D,E). These observations fit with the established mechanism of imipridones as CLPP agonists. We also observed an overall reduction in SMAD1/4 activity, which indicates that ONC206 treatment reduces SMAD signaling within the tumor microenvironment, pushing it towards a more cytostatic/differentiated state. The differences in effect observed in some cell models may reflect cell line-based heterogeneity and, therefore, are representative of their response to ONC206 treatment.

Altogether, the proteomics data from PDX and in vitro models clearly show that ONC206 works in UM by impairing mitochondrial function. To cope with this, the tumor cells seek an alternate energy source through lipid metabolism, as seen from both proteomics and metabolomics profiling.

## 4. Discussion

Metastatic UM remains a disease with profound unmet clinical need, driven in part by the lack of broadly effective systemic therapies. Building on our prior report that UM exhibits a strong dependence on mitochondrial OXPHOS, this study focused on a therapeutic strategy by testing the imipridone ONC206, which is currently being evaluated in multiple clinical studies. The recent FDA approval of the imipridone ONC201 for midline gliomas provides support for evaluation of the imipridones as a potentially clinically actionable strategy in other rare cancers.

This study is the first to evaluate the effects of the imipridone compound ONC206 on OXPHOS, cell survival, and the metabolomic and proteomic profiles of UM cells. We also examined the impact of ONC206 treatment on tumor growth and survival in UM PDX models in mice. We found that ONC206 treatment inhibits mitochondrial metabolism, strongly reduces OXPHOS both in primary and metastatic UM cell lines, and targets OXPHOS effectors and metabolites. The inhibition of SDHA/B was significant in most cell lines, with the exception of MEL20-06-039 (primary cell line), which showed a modest effect. ONC206 induces apoptosis and/or autophagy in UM cells depending on the cell line tested (irrespective of the primary or metastatic origin), inhibits tumor growth, and improves survival of preclinical models of UM. ONC206 may also promote UM cell differentiation, which may in turn lead to reduced tumor growth, similar to observations reported in diffuse midline glioma [29].

Interestingly, some UM cells (primary cell line MEL20-06-039 and metastatic cell line MM28) that demonstrated apoptotic responses to ONC206 also showed basal expression of the autophagy marker LC3B (Figure 2A,C). This pattern may be indicative of subclonal heterogeneity or simultaneous engagement of autophagic, apoptotic, and other cell death pathways, such as necrosis. Our data indicate that cell death mechanisms in response to ONC206 are context-dependent and may be shaped by intrinsic differences in metabolic and/or genetic dependencies. The increase in LC3B levels and autophagosome formation in some cells (Figure 2C) suggests that, in addition to apoptosis, autophagy may serve as a major stress response to ONC206-mediated mitochondrial disruption.

Metabolomic profile analyses also demonstrate a targeted reduction in OXPHOS metabolites and a change towards alternate energy metabolism pathways following ONC206 treatment. This metabolic reprogramming corresponds to ONC206-specific targeting of mitochondrial OXPHOS, accompanied by modulations in other cellular functions. For example, the heatmap of significantly altered metabolites reveals dose-dependent metabolic reprogramming to reduced OXPHOS levels and an increase in other energy metabolism pathways like fatty acid oxidation (Figure 4C). ONC206 also caused reprogramming of UM cell lipid metabolism, as seen in incomplete fatty acid oxidation, membrane remodeling, and PUFA accumulation. These responses to ONC206 ultimately induced oxidative stress-mediated cell death pathways. The dose-dependence seen in both protein and metabolite data indicates that the anti-tumor effects result from a systematic disruption of mitochondrial energy metabolism, confirming OXPHOS as a therapeutic target in UM.

As PDX models represent patient tumors considerably, we generated a new PDX model from a liver metastatic UM patient. We used this and another PDX model from the Wistar Institute to evaluate ONC206. ONC206 treatment significantly delayed tumor growth and improved survival in both PDX models. This strong in vivo response supports the therapeutic potential of ONC206 in UM. The proteomic profiling of UM-PDX tumors indicates that ONC206 simultaneously suppresses Src/RTK/YAP-driven mitogenic signaling and mitochondrial–redox homeostasis, forcing tumor cells into a vulnerable state with reduced capacity for proliferation. Surviving cells exhibit adaptive molecular mechanisms, including Akt signaling, lipid metabolism, and adhesion/communication pathways. The coordinated down-regulation of MAPK, PI3K–Akt, and YAP pathways suggests a collapse of upstream mitogenic and survival signaling.

In the present study, we observe broadly consistent trends in survival outcomes and multi-omics profiles with ONC206, comparable to those reported in our prior work with ONC212 [17]. Similarly, responses to ONC206 were observed in both primary and metastatic UM cell lines. However, extending beyond these shared features, our current analysis delineates the heterogeneous nature of cell death pathways induced by ONC206 and identifies adaptive survival mechanisms that may be leveraged to inform rational combination therapeutic strategies.

A limitation of this study is that ONC206 remains under clinical development and has not yet been approved by the U.S. FDA for cancer treatment. Hence, its safety profile and therapeutic efficacy in patients have not been fully established. However, it was well tolerated in our preclinical studies, and our findings are encouraging, providing a strong scientific rationale for continued evaluation of ONC206 and for recruiting UM patients into ongoing and future ONC206 clinical trials. Our current study positions ONC206 within a growing body of work that opens the possibility of clinical testing of ONC206 in OXPHOS-dependent malignancies not limited to UM [30,31,32,33].

Future research should investigate effective combination strategies that block adaptive survival pathways, such as autophagy, antioxidant defenses, lipid peroxidation, or Akt signaling, to enhance the depth and durability of the antitumor response.

## 5. Conclusions

Metastatic UM has very few treatment options and HLA-independent new therapeutic strategies are necessary for broad applicability. We demonstrate that ONC206 exerts potent antitumor activity in UM through the suppression of mitochondrial metabolism and modulation of metabolomic, lipidomic, and proteomic profiles. Together, our findings support a rationale for further testing of ONC206 for the treatment of metastatic UM, and potentially for other cancers that depend upon OXPHOS for survival.

## Data Availability

All relevant data are available from the corresponding author upon request.

## Supplementary Materials

The following supporting information can be downloaded at: https://www.mdpi.com/article/10.3390/cancers18121895/s1, Figure S1: CLPP as ONC206 target in UM cells; Figure S2: Quantitation of cleaved PARP; Figure S3: Heatmap of Mass spectrometry-based global metabolomics; Figure S4: Heatmap of changes in metabolism; Figure S5: Heatmap showing changes in global lipid profile; Figure S6: ONC206 treatment reduces tumor burden and improves survival of UM PDX model WM4481-1 in mice; Figure S7: Low magnification representative images of IHC analysis; Figure S8: Original western blot images for Figure 1B; Figure S9: Original western blot images for Figure S1A; Figure S10: Original western blot images for Figure 2; Figure S11: Original western blot images for Figure S6D; Figure S12: Original western blot images for Figure 5G.
