# Supplementary material for: The Imipridone ONC206 Inhibits Tumor Growth and Improves Survival in Patient-Derived Xenograft Models of Uveal Melanoma
Source: Cancers (Basel). 2026 Jun 10;18(12):1895. doi: 10.3390/cancers18121895 (PMC13296402; doi:10.3390/cancers18121895)
Supplement: Supplementary file 1 [file cancers-18-01895-s001.zip › cancers-4310651-supplementary.pdf]

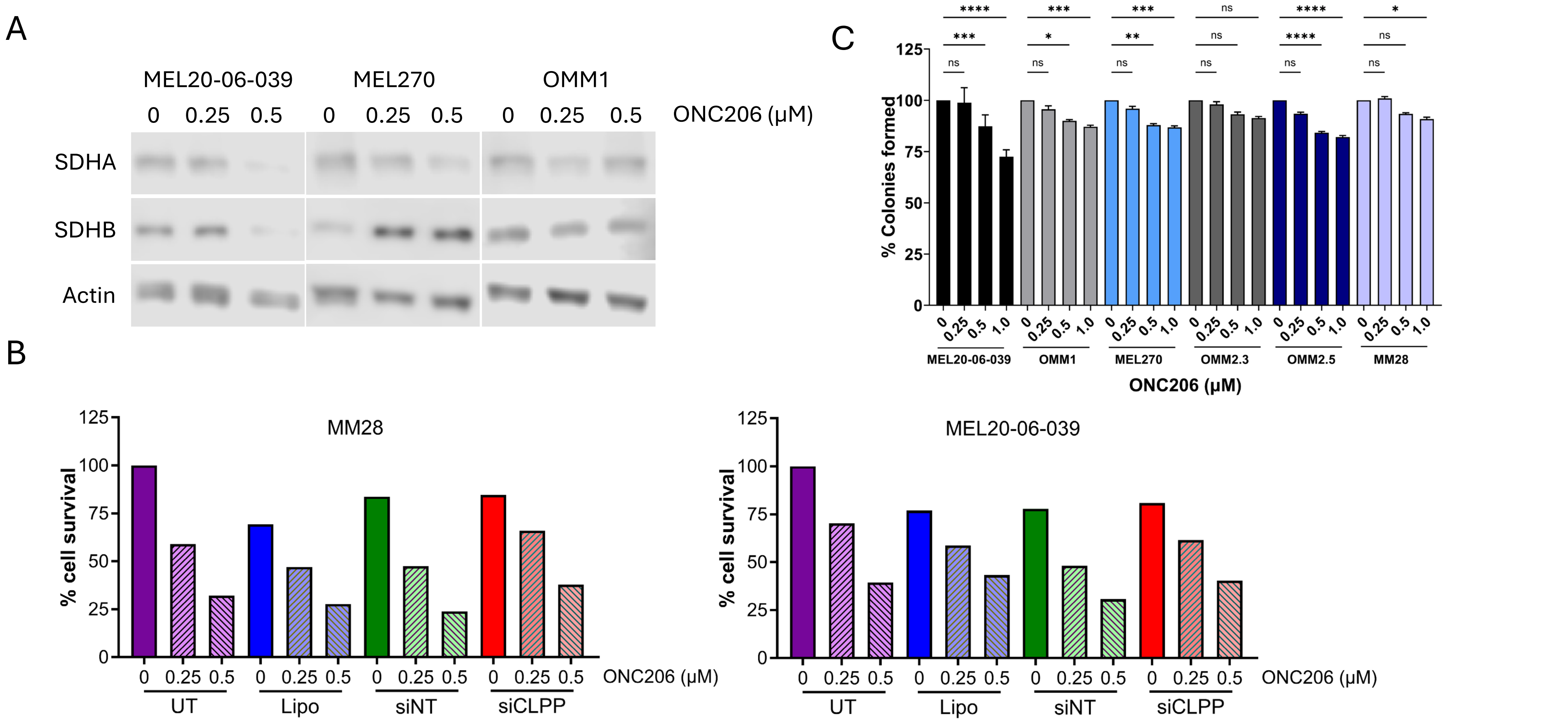

Figure S1: CLPP as ONC206 target in UM cells. (A) Western blots showing the levels of SDHA and SDHB post ONC206 treatment for 8 h in UM cell lines. (B) Cell viability assay post-CLPP knockdown and ONC206 treatment (0.25 &0.5 μM). UT, untreated; Lipo, lipofectamine; siNT, non-targeting control siRNA; siCLPP, CLPP siRNA. (C) Quantification of colony formation using imaging software (ImageJ). Stained colonies in each well were analyzed, and the results are presented as a bar graph.

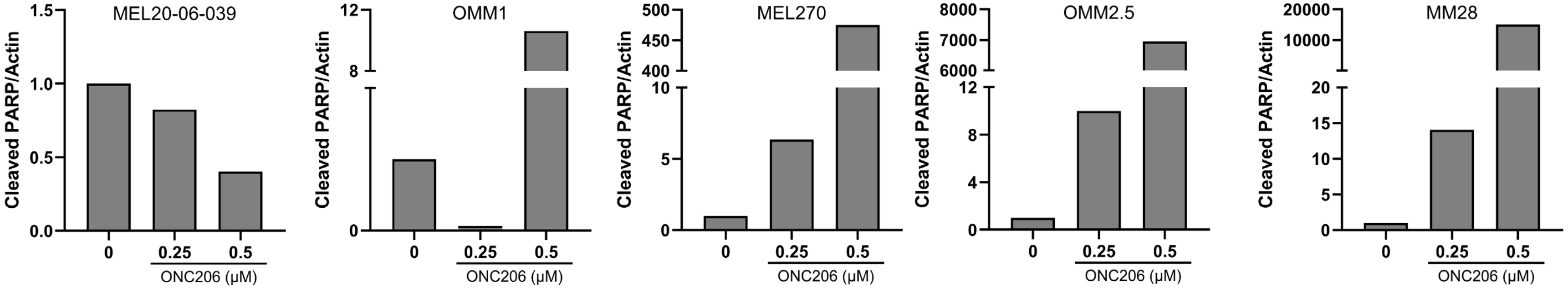

Figure S2: Quantitation of cleaved PARP expression levels from the western blots in Figure 2A; actin was used for normalization

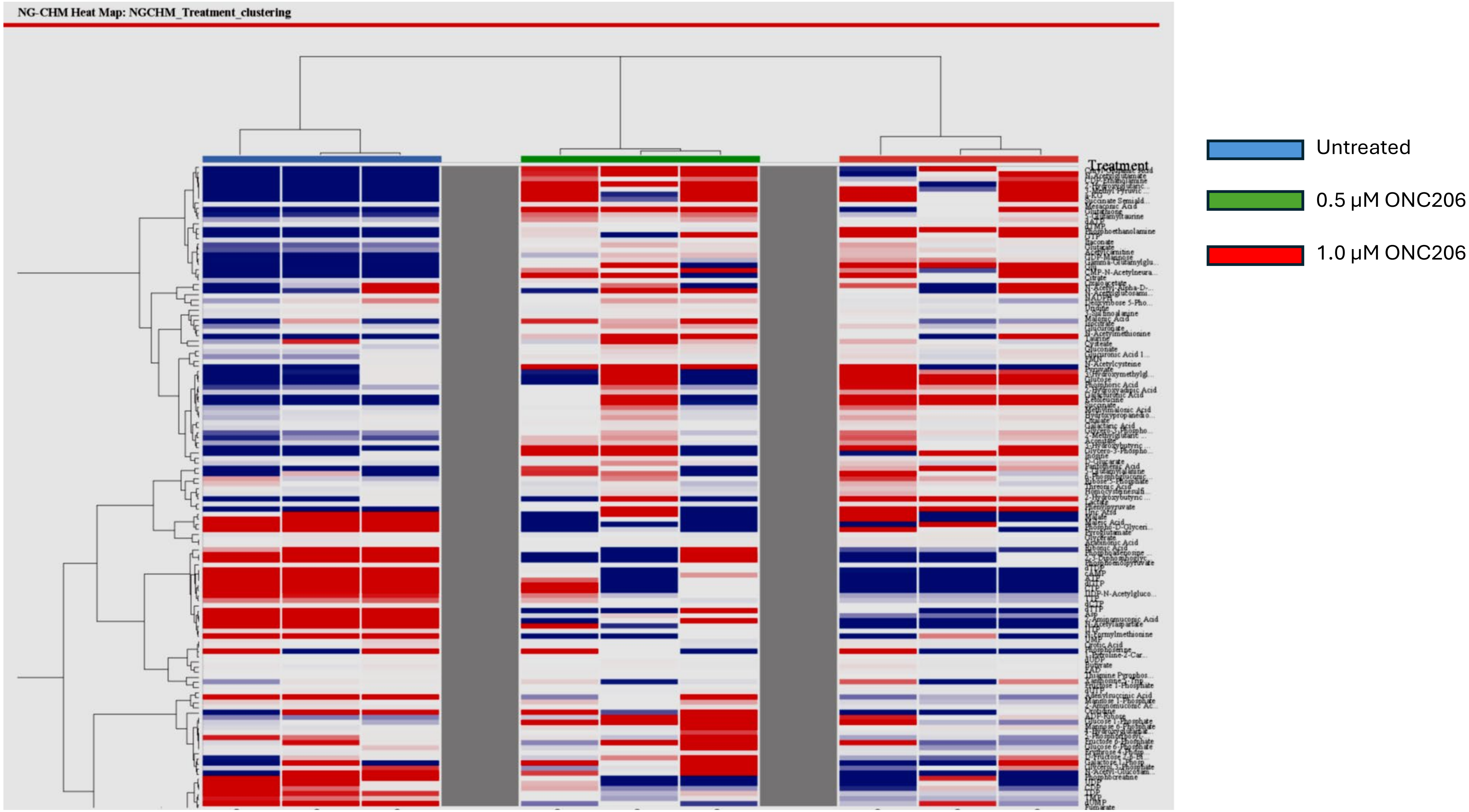

Figure S3: Heatmap of Mass spectrometry-based global metabolomics of UM cell line MM28, treated with 0.5 and 1 µM of ONC206 for 24 h shows multiple significant changes in metabolite profile.

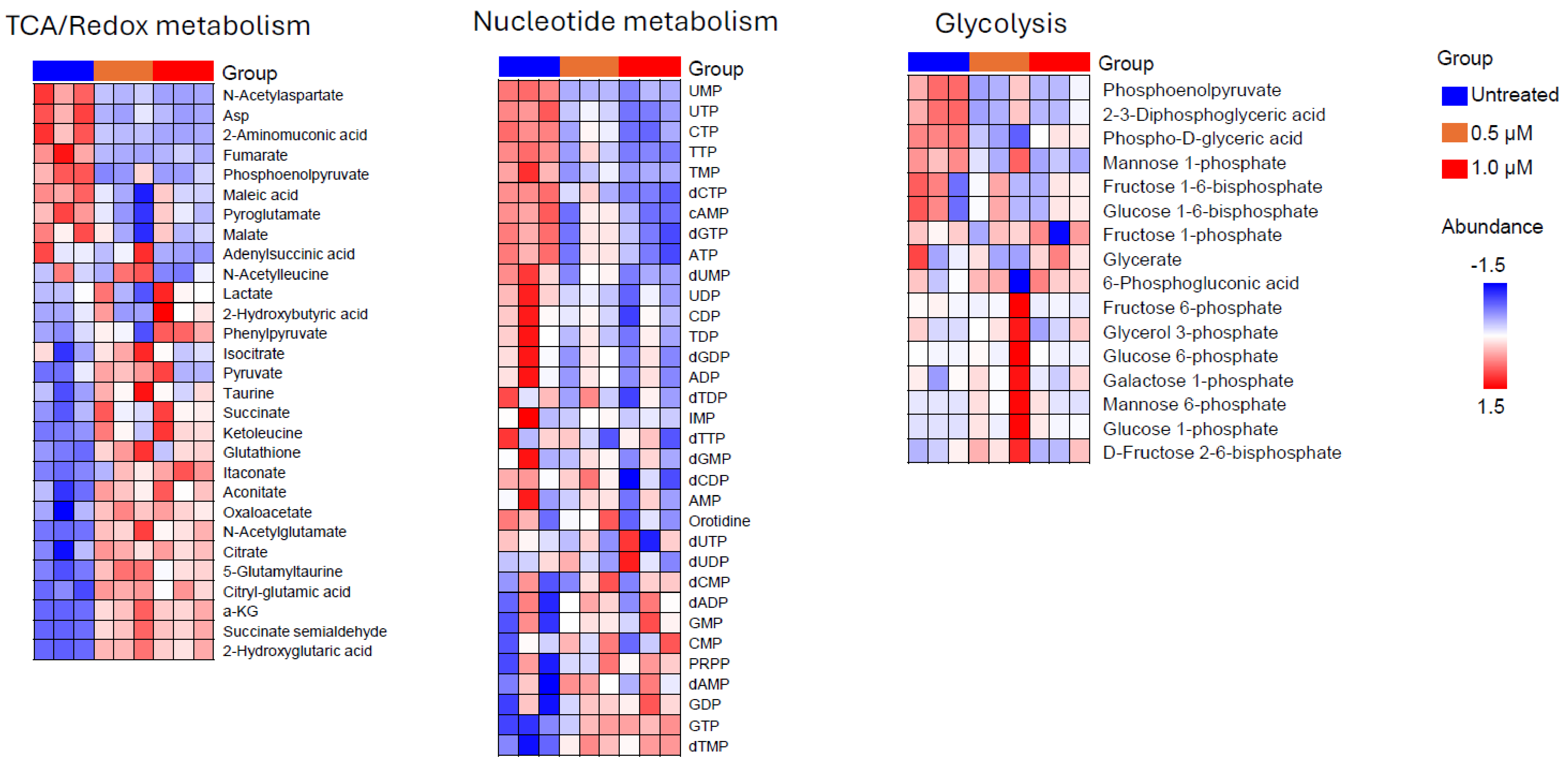

Figure S4: Heatmap of changes in TCA/Redox metabolism, nucleotide metabolism and Glycolysis related metabolites after treatment of MM28 cells with 0.5 and 1.0 μM of ONC206 for 24h.

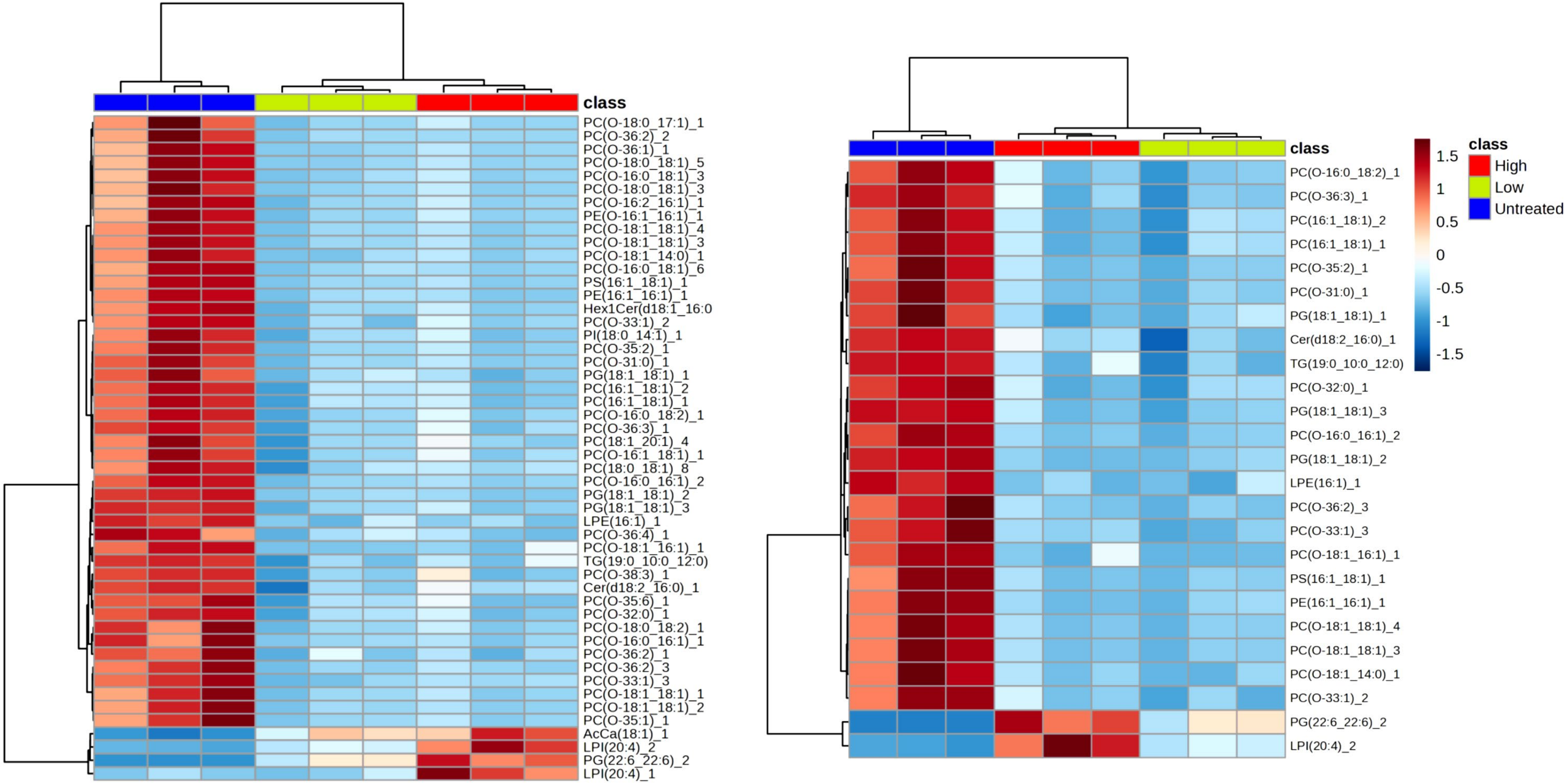

Figure S5: Heatmap showing changes in global lipid profile in MM28 cells after treatment with 0.5 and 1.0 µM ONC206 for 24h.

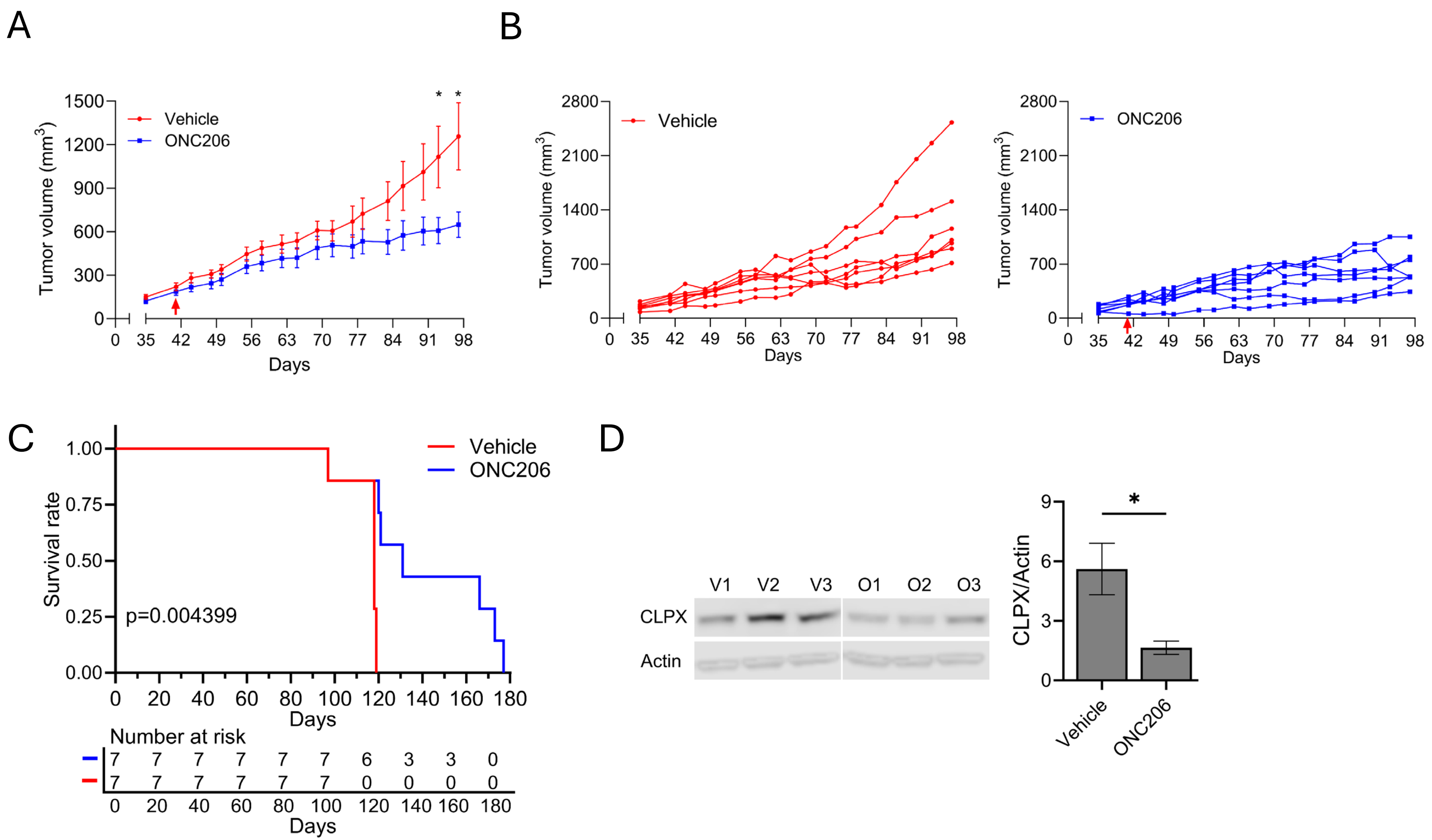

Figure S6: ONC206 treatment reduces tumor burden and improves survival of UM PDX model WM4481-1 in mice. **A.** Tumor growth curves in ONC206 and vehicle treated mice, plotted from 2X weekly tumor volume measurement post-tumor initiation. The red arrow indicates the beginning of ONC206 treatment. **B.** Individual mouse tumor growth plots in vehicle treated (left) and ONC 206 treated (right) mice, red arrow indicates beginning of treatment. **C.** Kaplan-Meier plots of WM4481-1 PDX model with ONC206 treatment compared to vehicle treated controls; n = 7 per treatment group; vehicle vs. ONC206 (p = 0.004399). **D.** Western blot analysis and quantitation of ONC206 treatment biomarker CLPX in three vehicle treated (V1-V3) and three ONC206 treated (O1-O3) mice tumors at week 6.

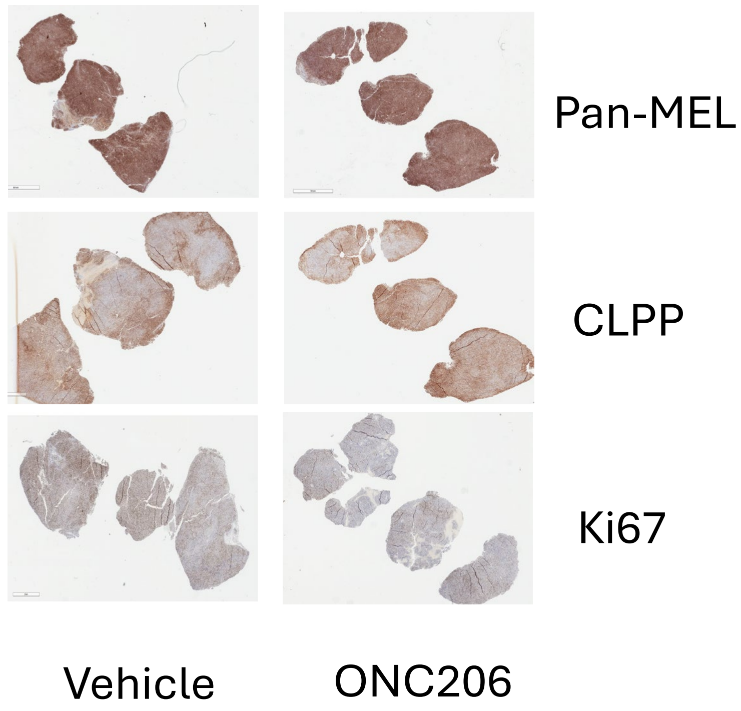

Figure S7: Low magnification representative images (scale bar=3mm) of Pan-MEL (melanoma), CLPP, and Ki67 immunohistochemical staining in vehicle and ONC206-treated tumors.

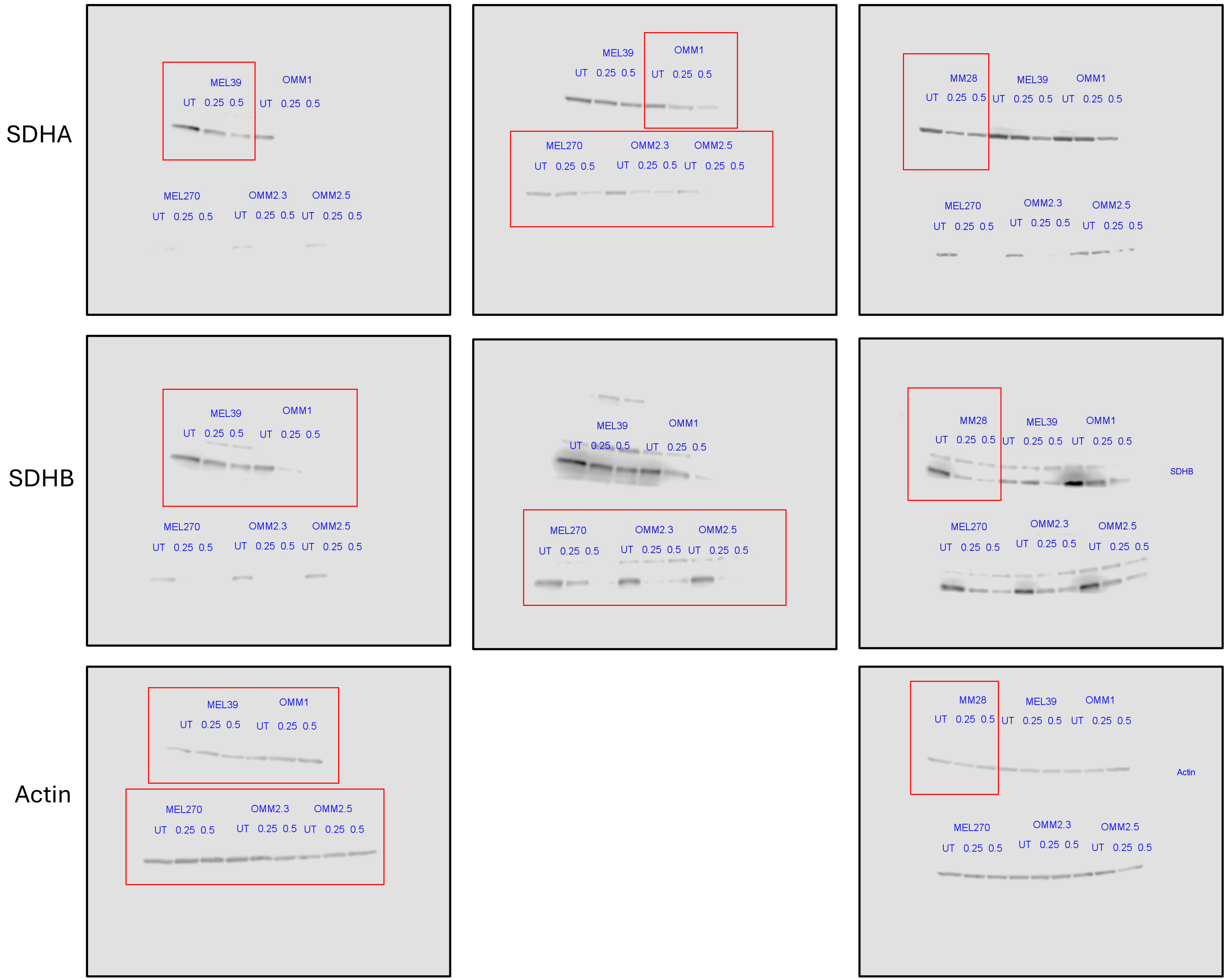

Figure S8: Original western blot images for Figure 1B

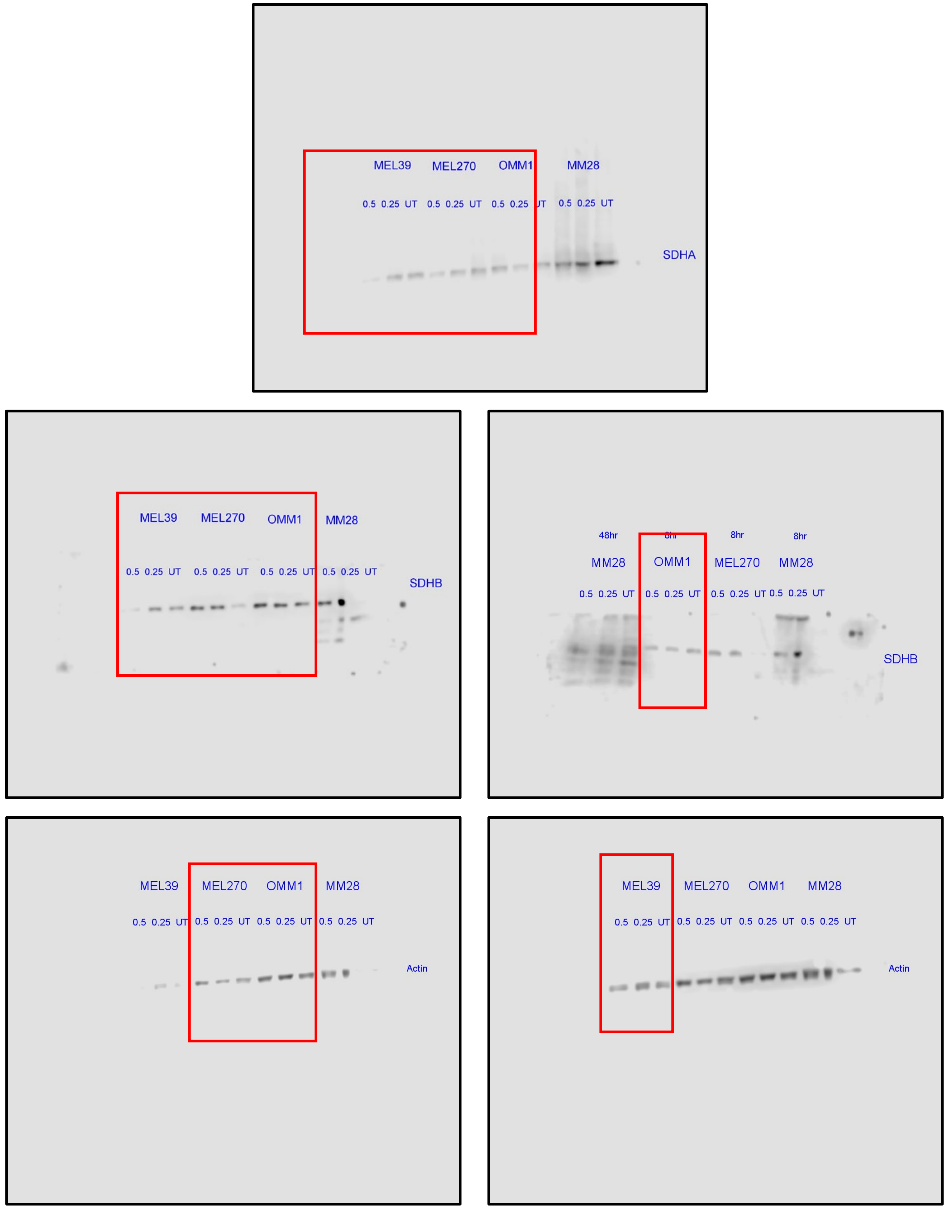

Figure S9: Original western blot images for figure S1A

Figure 2A

Cleaved PARP

Actin

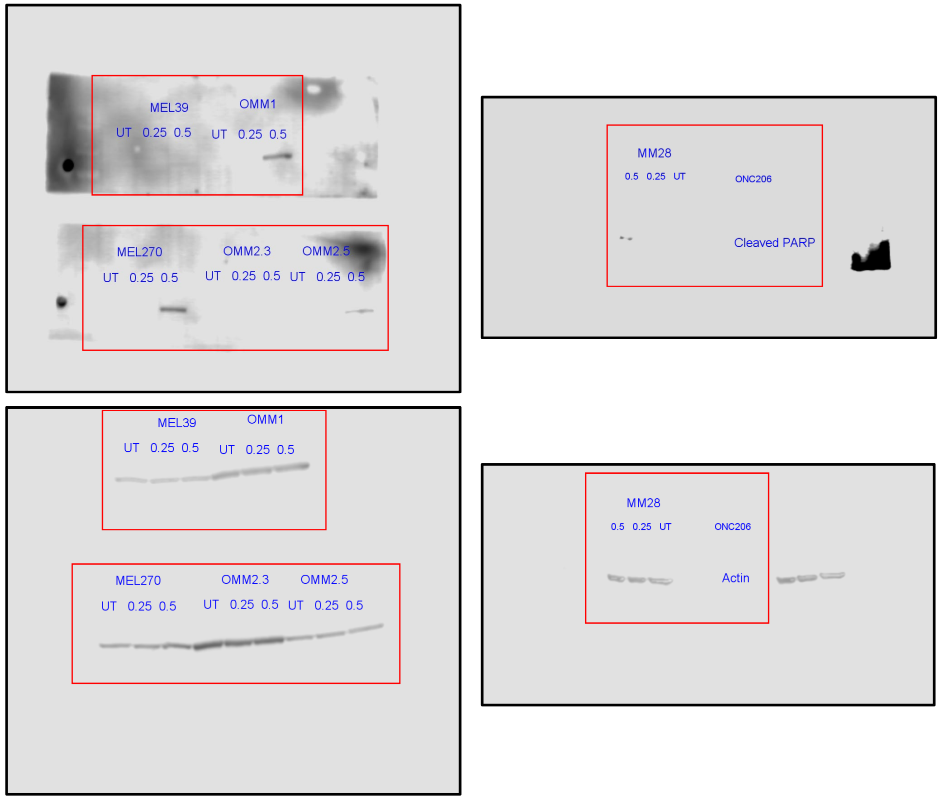

Figure 2B

LC3B

Actin

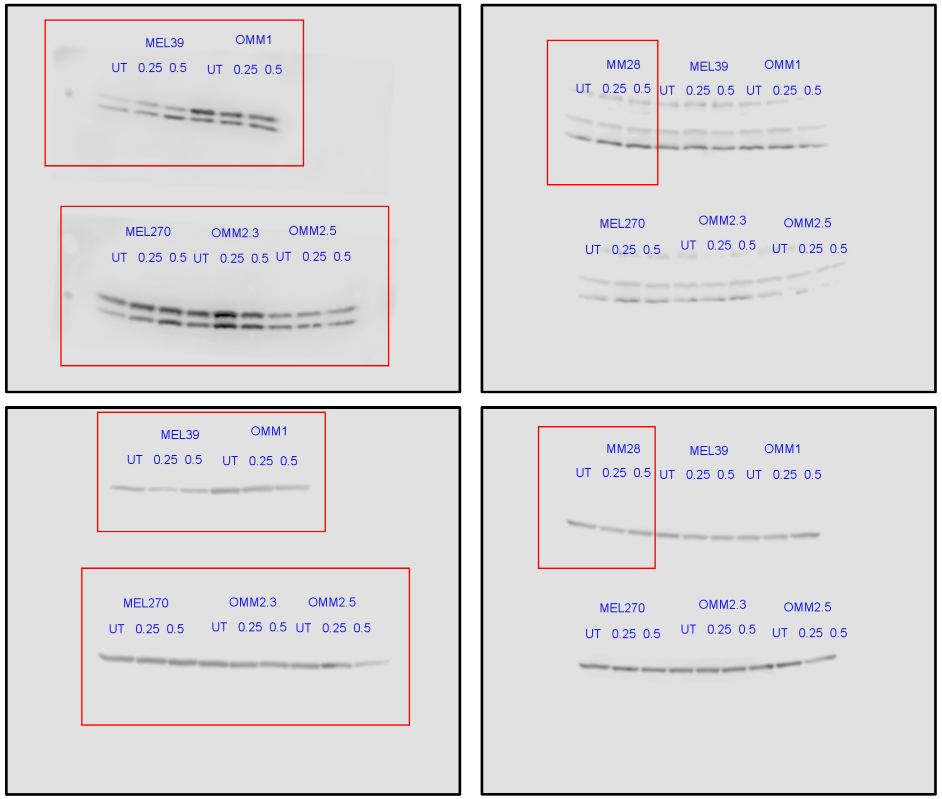

Figure S10: Original western blot images for Figure 2

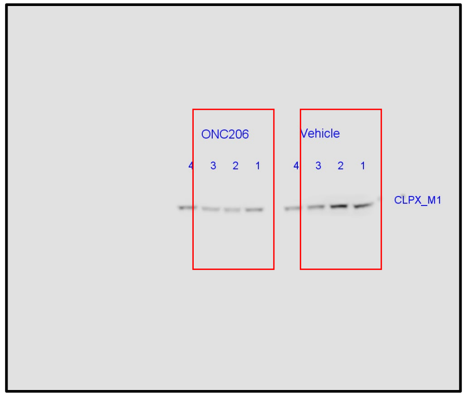

Lane	Samples
1	V3
2	V2
3	V1

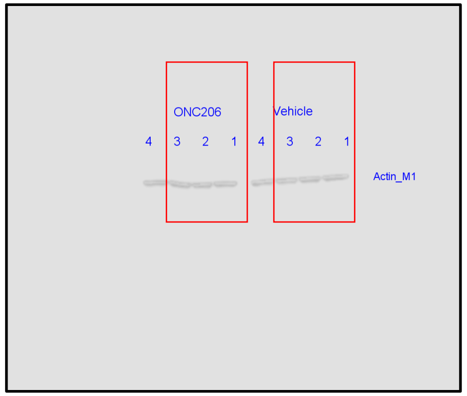

Lane	Samples
1	O3
2	O2
3	O1

Figure S11: Original western blot images for figure S6D

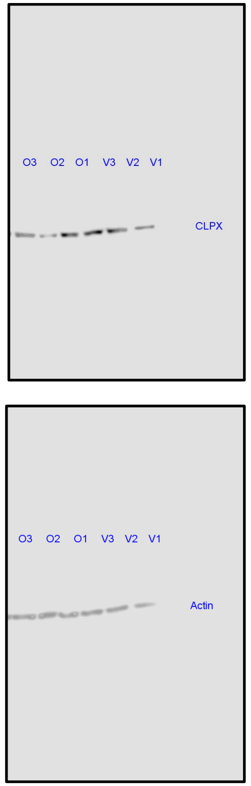

Figure S12: Original western blot images for Figure 5G
